# Excess mortality in Poland during the first and second wave of the COVID-19 pandemic in 2020

**DOI:** 10.3389/fpubh.2022.1048659

**Published:** 2022-11-17

**Authors:** Małgorzata Pikala, Małgorzata Krzywicka, Monika Burzyńska

**Affiliations:** ^1^Department of Epidemiology and Biostatistics, The Chair of Social and Preventive Medicine of the Medical University of Lodz, Łódź, Poland; ^2^Faculty of Technical Physics, Information Technology, and Applied Mathematics, Lodz University of Technology, Łódź, Poland

**Keywords:** excess mortality, COVID-19, causes of death, mortality trends, estimation, Poland

## Abstract

**Purpose:**

The aim of the study was to analyse excess deaths by major causes of death and associated changes in the mortality pattern of the Polish population in 2020 due to the impact of the COVID-19 pandemic.

**Methods:**

The study used data on all deaths in Poland which occurred between 2010 and 2020 (*N* = 3,912,237). 10-year mortality trends for 2010–2019 were determined. An analysis of time trends has been carried out with joinpoint models and Joinpoint Regression Program. Based on the determined regression models, the number of deaths expected in 2020 and the number of excess deaths due to selected causes were calculated.

**Results:**

The crude death rates of all-cause deaths increased from 2000 to 2019 at an average annual rate of 1% (*p* = 0.0007). The determined regression model revealed that the number of deaths in 2020 should have been 413,318 (95% CI: 411,252 to 415,385). In reality, 477,355 people died in Poland that year. The number of excess deaths was therefore 64,037 (15.5%). According to data from Statistics Poland the number of COVID-19-related deaths was 40,028, the number of non-COVID-19 deaths was 24,009. The largest percentage increase over the expected number of deaths was observed for suicide (12.5%), mental and behavioral disorders (7.2%) and diseases of circulatory system (5.9%). A lower than expected number of deaths was observed for malignant neoplasms (−3.2%) and transport accidents (−0.1%).

**Conclusion:**

The difference between expected and observed non-COVID-19 deaths in 2020 indicates a need for further analysis of the causes of excess mortality.

## Introduction

In Poland, the first case of SARS-CoV-2 virus was detected on 4 March 2020. It is assumed that there were two waves of the announced COVID-19 epidemic in 2020. It is difficult to clearly determine the boundary between different waves of the coronavirus infection. Two parameters are crucial: the reproduction rate of the virus (R) and the actual increment in the infection rate. The time when R exceeds 1 and begins to rise, which entails an increase in the number of infections, is considered the onset of the wave. Conversely, when R falls below 1, the epidemic begins to slow down. The development of the first wave was successfully halted in Poland by introducing a long lockdown. This resulted in daily increases in infections being highly dragged out and not very high. The onset of the second wave is conventionally assumed to have occurred in the middle of September 2020, while the peak of the second wave is assumed to have occurred at the beginning of November 2020 (1).

In 2020, there were 477,355 all-cause deaths, which meant that the crude death rate (CDR) was 1247.5 per 100,000 population. Compared to 2019, the number of deaths increased by 67,646. According to recent reports, that was the highest number of deaths since the World War II (2). Data from Statistics Poland show that 40,028 people died of COVID-19 in Poland in 2020 (CDR = 104.6 per 100,000 population). This implies that more than 27,000 deaths from causes other than COVID-19 were reported (3).

The WHO defines excess mortality as, “the mortality above what would be expected based on the non-crisis mortality rate in the population of interest.” Excess deaths not attributed to COVID-19 could include: deaths from other causes resulting from inadequate or lack of any access to healthcare services for non-COVID-19-related diseases, deaths from other causes resulting from changes in risk factors or the prevalence of certain illness or injuries and deaths that are actually related to COVID-19 but not recorded as such. Furthermore, overwhelmed health systems and stretched capacity could potentially result in substandard management of non-COVID-19-related conditions, exacerbating pre-existing shortages and inequities contributing to excess mortality (4).

A study of 29 high-income countries revealed that Poland ranked among the top five countries with the highest absolute number of excess deaths, after the United States, Italy, England and Wales and Spain (3). Per 100,000 population, the crude excess death rate for men was the second highest after Lithuania among the analyzed countries. The situation was slightly better in the group of women, where the crude excess death rate was the eighth highest value among the above-mentioned 29 countries (5).

The aim of the study was to analyse excess deaths by the main causes of death and associated changes in mortality patterns in Poland in 2020 caused by the impact of the COVID-19 pandemic. Excess deaths were defined in this study as the difference between the actual number of deaths in 2020 and the expected number of deaths, estimated with the use of regression models determined for the previous 10 years, i.e., for the period 2010–2019. The analysis will allow us to determine for which group of diseases, being the underlying cause of death, the observed number of deaths differed most from the number of expected deaths.

## Materials and methods

The study used data on all deaths of Polish residents which occurred between 2010 and 2020 (*N* = 3,912,237). The database was created from death certificates collected and made available for this study by Statistics Poland.

The study identifies deaths from COVID-19 (according to the International Statistical Classification of Diseases and Health Related Problems – Tenth Revision – ICD-10, coded as U07.1). In addition, the analysis included five most common major groups of causes of death: diseases of the circulatory system (I00–I99), malignant neoplasms (C00–C97), diseases of the respiratory system (J00–J99), diseases of the digestive system (K00–K93) as well as external causes of mortality, of which two main groups were identified: transport accidents (V01–V99) and suicide (X60–X84). Due to very rapidly increasing mortality rates and the importance of these causes during the pandemic period, mental and behavioral disorders (F00–F99) were also included in the analysis.

Crude death rates (CDR) were calculated according to the following formula:


$$\begin{array}{l} CDR=\frac{k}{p}*100,000 \end{array}$$

where : k – number of deaths; p –population size.

10-year mortality trends for the period 2010–2019 have been determined. The analysis of time trends has been carried out with joinpoint models and Joinpoint Regression Program, a statistical software package developed by the U.S. National Cancer Institute for the Surveillance, Epidemiology and End Results Program (6).

Joinpoint regression model is an advanced version of linear regression *y* = *x'beta* + *e*, where: beta is regression coefficient, e is the y-intercept, y is a measure evaluated in the study and x is a calendar year. Time trends were determined with the use of segments joining in joinpoints where trend values significantly changed (*p* < 0.05). To confirm whether the changes were statistically significant, the Monte Carlo Permutation method was applied.

In addition, the authors also calculated Annual Percentage Change (APC) for each segments of broken lines and average annual percentage change (AAPC) for the whole study period with corresponding 95% confidence intervals (CI).

Annual Percent Change is used to characterize trends in death rates over time and it was calculated according to the following formula:


$$\begin{array}{l} APC=100*\left(\exp^{b}-1\right)\; \end{array}$$

where: b – the slope coefficient.

Average Annual Percent Change (AAPC) is a summary measure of the trend over a pre-specified fixed interval. It allows us to use a single number to describe the average APCs over a period of multiple years. It is valid even if the joinpoint model indicates that there were changes in trends during those years. It is computed as a weighted average of the APCs from the joinpoint model, with the weights equal to the length of the APC interval (7).


$$AAPC=\left\{\exp\left(\frac{\sum w_{i}b_{i}i}{\sum w_{i}}\right)-1\right\}\;\times \;100$$

where: *b*_*i*_ - the slope coefficient for each segment in the desired range of years, *w*_*i*_ - the length of each segment in the range of years.

Statistic difference of APC and AAPC was assessed using the Z test, the terms “increase” or “decrease” describing a statistically significant (*p* < 0.05) APC and AAPC, and “stable” for non-significant trends.

Based on the determined regression models, the number of deaths expected in 2020 and the number of excess deaths due to selected causes were calculated according to the following formula:

number of excess deaths = number of deaths observed - number of deaths expected.

For a trend which was statistically insignificant, the arithmetic mean for the years 2017–2019 was identified with the number of expected deaths. This information was given in each case in the text and in the table presenting results.

Statistical analyses were performed using the Statistica (data analysis software system), version 13 (TIBCO Software Inc.).

## Results

**Figure 1:**
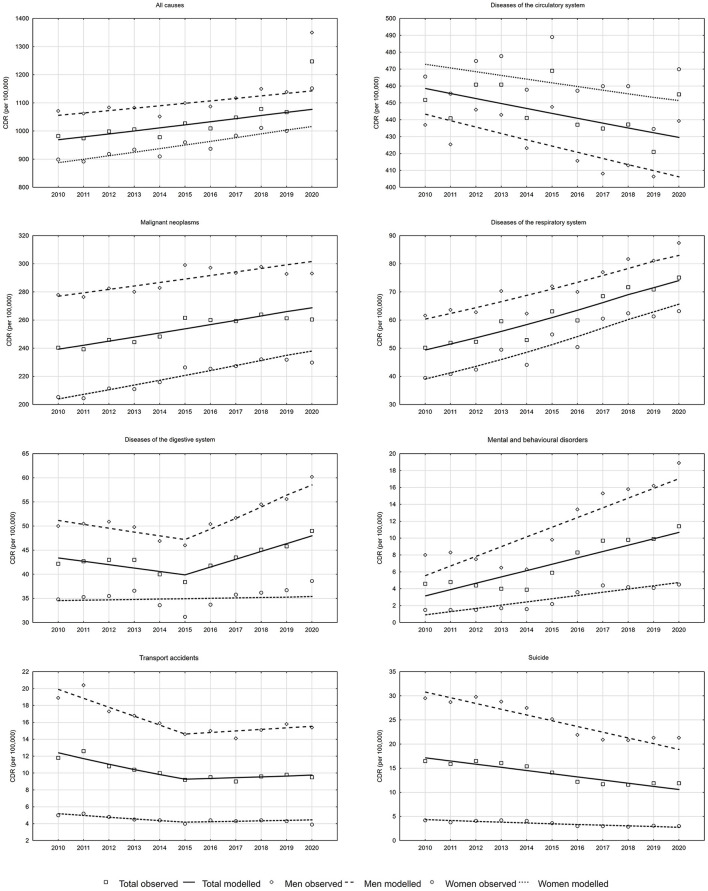
Trends in crude death rates due to all causes and due to selected causes of death in the years 2010–2020 in Poland.

**Figure 2:**
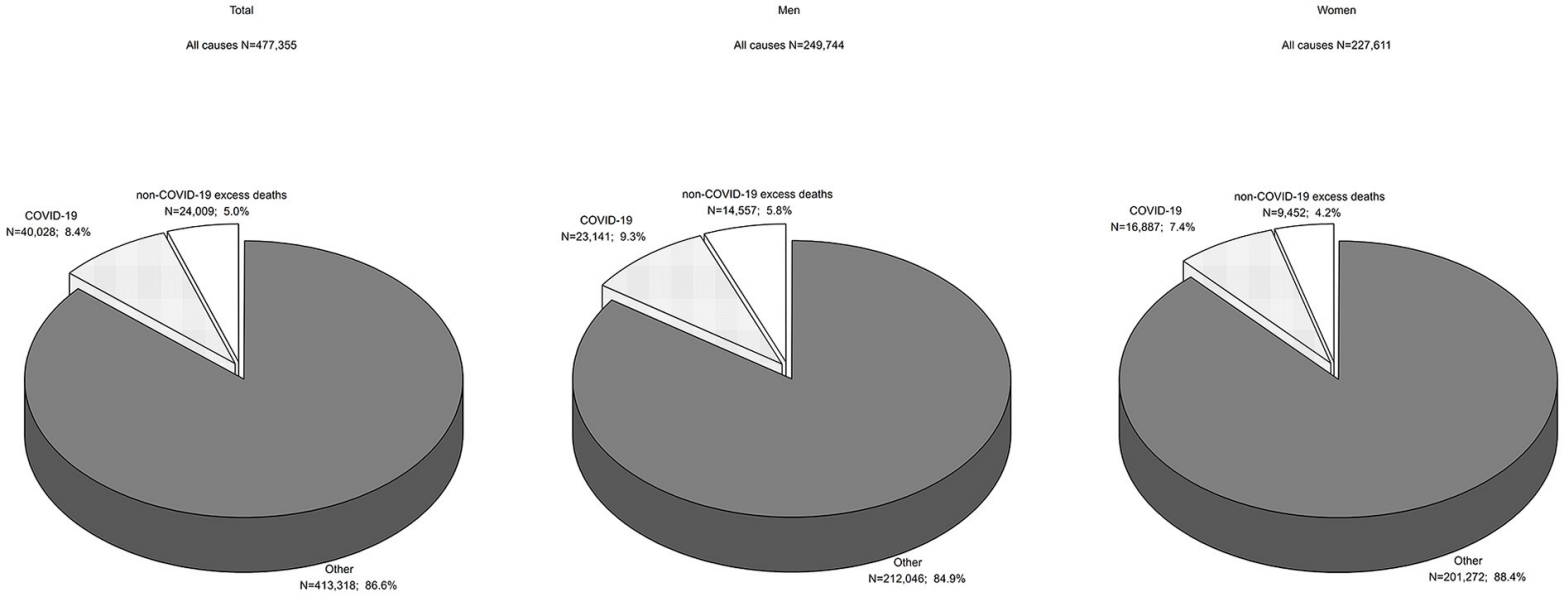
Excess mortality in 2020 due to COVID-19 and non-COVID-10 causes in total and by gender.

The number of all-cause deaths in Poland increased from 378,478 in 2010 to 409,709 in 2019 (Table 1). The crude death rate (CDR) in 2010 was 982.3 per 100,000 population. In 2019, it increased to 1067.4 per 100,000 population (Figure 1). The Annual Percentage Change (APC) value between 2010 and 2019 was 1.0 and it was high significance (*p* = 0.0007, Table 2). According to a determined regression model, the number of deaths in 2020 should have been 413,318. In fact, 477,355 people died in that year in Poland (Table 1), and the CDR was 1247.5 per 100,000 population (Figure 1). The number of excess deaths was therefore 64,037 (15.5%) (Table 2). According to data from Statistics Poland due to COVID-19, 40,028 people died in 2020 in Poland, which accounted for 62.5% of all excess deaths (CDR = 104.6 per 100,000 population). This shows that 24,009 excess deaths (i.e., 37.5%) appeared to have occurred due to causes other than COVID-19 (Figure 2).

**Table 1:** Total number of deaths and due to selected causes in 2010–2020 in Poland.

**Year**	**2010**	**2011**	**2012**	**2013**	**2014**	**2015**	**2016**	**2017**	**2018**	**2019**	**2020**
**Total**											
All causes	378,478	375,501	384,788	387,312	376,467	394,921	388,009	402,852	414,200	409,709	477,355
Diseases of the circulatory system (I00–I99)	173,972	169,846	177,534	177,387	169,698	180,343	167,974	167,075	167,942	161,589	174,546
Malignant neoplasms (C00–C97)	92,609	92,196	94,739	94,116	95,565	100,601	99,959	99,643	101,386	100,323	99,867
**Diseases of the respiratory system (J00–J99)**	19,342	19,982	20,148	22,947	20,371	24,279	23,013	26,309	27,561	27,219	28,732
Diseases of the digestive system (K00–K93)	16,231	16,434	16,559	16,536	15,394	14,756	16,044	16,719	17,309	17,597	18,808
Transport accidents (V01–V99)	4,529	4,835	4,170	4,010	3,837	3,525	3,655	3,477	3,672	3,776	3,638
Suicide (X60–X84)	6,342	6,112	6,365	6,215	5,933	5,417	4,671	4,482	4,441	4,567	4,553
Mental and behavioral disorders (F00–F99)	1,784	1,859	1,708	1,546	1,495	2,268	3,204	3,723	3,762	3,807	4,387
**Men**											
All causes	199,833	198,178	202,094	201,696	195,791	204,469	202,150	207,671	213,647	211,423	249,744
Diseases of the circulatory system (I00–I99)	81,474	79,338	83,169	82,514	78,808	83,288	77,294	75,861	76,754	75,473	81,489
Malignant neoplasms (C00–C97)	51,816	51,552	52,699	52,197	52,690	55,663	55,250	54,559	55,361	54,370	54,367
**Diseases of the respiratory system (J00–J99)**	11,498	11,872	11,715	13,106	11,611	13,390	13,021	14,312	15,186	15,067	16,220
Diseases of the digestive system (K00–K93)	9,322	9,424	9,497	9,272	8,726	8,555	9,362	9,616	10,134	10,320	11,169
Transport accidents (V01–V99)	3,531	3,808	3,220	3,124	2,965	2,723	2,784	2,630	2,806	2,928	2,856
Suicide (X60–X84)	5,506	5,352	5,555	5,375	5,122	4,695	4,075	3,881	3,870	3,957	3,953
Mental and behavioral disorders (F00–F99)	1,489	1,557	1,407	1,218	1,181	1,828	2,489	2,851	2,932	3,002	3,500
**Women**											
All causes	178,645	177,323	182,694	185,616	180,676	190,452	185,859	195,181	200,553	198286	227,611
Diseases of the circulatory system (I00–I99)	92,498	90,508	94,365	94,873	90,890	97,055	90,680	91,214	91,188	86,116	93,057
Malignant neoplasms (C00–C97)	40,793	40,644	42,040	41,919	42,875	44,938	44,709	45,084	46,025	45,953	45,500
**Diseases of the respiratory system (J00–J99)**	7,844	8,110	8,433	9,841	8,760	10,889	9,992	11,997	12,375	12,152	12,512
Diseases of the digestive system (K00–K93)	6,909	7,010	7,062	7,264	6,668	6,201	6,682	7,103	7,175	7,277	7,639
Transport accidents (V01–V99)	998	1,027	950	886	872	802	871	847	866	848	782
Suicide (X60–X84)	836	760	810	840	811	722	596	601	571	610	600
Mental and behavioral disorders (F00–F99)	295	302	301	328	314	440	715	872	830	805	887

**Table 2:** Excess deaths in 2020 in total and due to selected causes in Poland.

	**APC (95% CI)**	***p***	**AAPC 2010–2019 (95% CI)**	***p***	**Number of deaths expected in 2020 (95% CI)**	**Number of deaths observed in 2020**	**Number of deaths in excess in 2020**	**Excess mortality in 2020 (%)**
	**Total**							
All causes	1.0^*^ (0.6; 1.5)	0.0007	1.0^*^ (0.6; 1.5)	0.0007	413,318 (411,252; 415,385)	477,355	64,037	15.5
Diseases of the circulatory system (I00–I99)	−0.7^1^ (−1.4; −0.9)	0.0548	−0.7 (−1.4; −0.9)	0.0548	164,847 (163,693; 166,001)	174,546	9,699	5.9
Malignant neoplasms (C00–C97)	1.1^*^ (0.7; 1.5)	0.0001	1.1^*^ (0.7; 1.5)	0.0001	103,142 (102,729; 103,554)	99,867	−3,275	−3.2
Diseases of the respiratory system (J00–J99)	4.2^*^ (2.9; 5.6)	< 0.0001	4.2^*^ (2.9; 5.6)	< 0.0001	28,412 (28,043; 28,810)	28,732	320	1.1
Diseases of the digestive system (K00–K93)	3.9^2^^*^ (1.0; 6.8)	0.0084	0.8 (−0.5; 2.0)	0.0707	18,413 (17,879; 18,947)	18,808	395	2.1
Transport accidents (V01–V99)	1.0^2^ (−4.1; 6.4)	0.6438	−2.8^*^ (−5.1; −0.4)	0.0406	3,642^3^ (3,456; 3,835)	3,638	−4	−0.1
Suicide (X60–X84)	−4.7^*^ (−6.1; −3.2)	0.0001	−4.7^*^ (−6.1; −3.2)	0.0001	4,047 (3,990; 4,103)	4,553	506	12.5
Mental and behavioral disorders (F00–F99)	11.7^*^ (5.8; 17.9)	0.0015	11.7^*^ (5.8; 17.9)	0.0015	4,094 (3,852; 4,348)	4,387	293	7.2
	**Men**							
All causes	0.7^1^ (0.3; 1.2)	0.0049	0.7 (0.3; 1.2)	0.0049	212,046 (211,198; 212,894)	249,744	37,698	17.8
Diseases of the circulatory system (I00–I99)	−0.9^*^ (−1.6; −0.2)	0.0165	−0.9^*^ (−1.6; −0.2)	0.0165	75,403 (74,876; 75,931)	81,489	6,086	8.1
Malignant neoplasms (C00–C97)	0.8^*^ (0.4; 1.3)	0.0032	0.8^*^ (0.4; 1.3)	0.0032	55,985 (55,761; 56,209)	54,367	−1,618	−2.9
Diseases of the respiratory system (J00–J99)	3.3^*^ (2.0; 4.5)	0.0003	3.3^*^ (2.0; 4.5)	0.0003	15,405 (15,205; 15,590)	16,220	815	5.3
Diseases of the digestive system (K00–K93)	4.5^2^^*^ (1.9; 7.2)	0.0042	1.1 (−0.1; 2.2)	0.0637	10,863 (10,580; 11,156)	11,169	306	2.8
Transport accidents (V01–V99)	0.8^2^ (−6.1; 8.2)	0.4355	−2.4 (−5.7; 1.1)	0.1768	2,788^3^ (2,596; 2,994)	2,856	68	2.4
Suicide (X60–X84)	−4.7^*^ (−6.1; −3.3)	< 0.0001	−4.7^*^ (−6.1; −3.3)	< 0.0001	3,505 (3,456; 3,554)	3,953	448	12.8
Mental and behavioral disorders (F00–F99)	10.8^*^ (4.7; 17.1)	0.0029	10.8^*^ (4.7; 17.1)	0.0029	3,159 (2,967; 3,359)	3,500	341	10.8
	**Women**							
All causes	1.3^*^ (0.9; 1.8)	0.0002	1.3^*^ (0.9; 1.8)	0.0002	201,272 (200,467; 202,078)	227,611	26,339	13.1
Diseases of the circulatory system (I00–I99)	−0.5^1^ (−1.3; 0.3)	0.1741	−0.5 (−1.3; 0.3)	0.1741	89,506^3^ (88,890; 90,222)	93,057	3,551	4.0
Malignant neoplasms (C00–C97)	1.5^*^ (1.2; 1.9)	< 0.0001	1.5^*^ (1.2; 1.9)	< 0.0001	47,157 (47,015; 47,345)	45,500	−1,657	−3.5
Diseases of the respiratory system (J00–J99)	5.6^*^ (3.9; 7.2)	< 0.0001	5.6^*^ (3.9; 7.2)	< 0.0001	13,007 (12,796; 13,215)	12,512	−495	−3.8
Diseases of the digestive system (K00–K93)	0.2^1^ (−1.1; 1.5)	0.7321	0.2 (−1.1; 1.5)	0.7321	7,185^3^ (7,092; 7,278)	7,639	454	6.3
Transport accidents (V01–V99)	1.0^2^ (−2.1; 4.1)	0.4438	−1.9^*^ (−3.3; −0.5)	0.0154	854^3^ (827; 880)	782	−72	−8.4
Suicide (X60–X84)	−4.4^*^ (−6.4; −2.4)	0.0012	−4.4^*^ (−6.4; −2.4)	0.0012	542 (531; 552)	600	58	10.8
Mental and behavioral disorders (F00–F99)	15.7^*^ (10.0; 21.7)	0.0002	15.7^*^ (10.0; 21.7)	0.0002	934 (881; 991)	887	−47	−5.1

* p < 0.05,

1 APC 2010–2019,

2 APC 2015–2019,

3 Average for 2017–2019.

Among men, the APC value for all-cause deaths between 2010 and 2019 was 0.7% (*p* = 0.0049). The number of deaths expected in 2020 was 212,046 and the number of recorded deaths was 249,744 (Table 2). The number of excess deaths in the male group was 37,698 (17.8%). Of this number, COVID-19 contributed to 23,141 deaths (CDR = 125.1 per 100,000 men). The remaining 14,557 excess deaths occurred due to other causes (Figure 2).

Among women, CDRs increased from 898.8 in 2010 to 1,000.7 in 2019 (APC = 1.3%, *p* = 0.0002) (Figure 1). The regression model shows that 201,272 deaths were expected in 2020. In reality, 227,611 women died. The number of excess deaths was therefore 26,339 (13.1%) (Table 2) and COVID-19 contributed to 16,887 deaths (CDR = 85.4 per 100,000 women); in 9,452 death certificates, the causes of mortality were other than COVID-19 (Figure 2).

CDRs due to cardiovascular disease presented a decreasing trend between 2010 and 2019, but it was low significance (APC = −0.7%, *p* = 0.0548) (Table 2). In 2020, the CDR value increased to 455.1 per 100,000 population (Figure 1). Instead of 164,847 expected deaths, there were 174,546 deaths which occurred due to cardiovascular diseases. Thus, the difference between the expected and recorded deaths was 9,699 (5.9%) (Table 2).

Among men, between 2000 and 2019, the number of deaths due to cardiovascular diseases decreased at a rate of −0.9% (*p* = 0.0165). For this rate, the number of deaths in 2020 should have been 75,403. In fact, 6,086 more deaths (8.1%) occurred (Table 2). This resulted in an increase in CDRs values to 439.3 per 100,000 men (Figure 1). In the group of women, between 2000 and 2019, trend of CDRs was stable (*p* = 0.1741) (Table 2). The difference between the number of deaths recorded in 2020 and the average number of deaths recorded in the years 2017–2019 corresponded to a value which was 4.0 % higher than expected.

A lower than expected number of deaths due to malignant tumors was observed overall and in both gender groups. Between 2010 and 2019, malignant cancer mortality trends were increasing. APC was 1.1% (*p* = 0.0001) (0.8%, *p* = 0.0032 in the male group and 1.5%, *p* < 0.0001 in the female group). Instead of the expected 103,142 cancer deaths, there were 3,275 fewer, i.e., 99,867 (54,367 in the male group and 45,500 in the female group) (Table 2).

From 2000 to 2019, CDRs due to respiratory diseases in Poland increased at an average annual rate of 4.2% (*p* < 0.0001) (3.3%, *p* = 0.0003 in the male group and 5.6%, *p* < 0.0001 in the female group). According to the regression model, in 2020, the expected number of deaths from these causes should have been 28,412 (15,405 in the male group and 13,007 in the female group). In reality, there were 1.1% more deaths, i.e., 28,732. The increase was due to higher, than expected, male mortality (by 5.3%, i.e. 815 deaths). For women, there were 495 fewer deaths than expected (−3.8%) (Table 2).

With regards to digestive diseases, the trend changed in 2015 (Figure 1). In the years 2015–2019, APC was 3.9% (*p* = 0.0084), which means that the number of deaths expected due to these causes in 2020 was 18,413. Eighteen thousand eight hundred and eight people actually died, which indicates that the number of excess deaths was 395 (2.1%). In the group of men, the CDR value increased from 46.0 in 2015 to 55.6 in 2019 (Figure 1) (APC = 4.5%, *p* = 0.0042). In 2020, the number of deaths expected in the male group was 10,863. The number of deaths which actually occurred was 2.8% higher, i.e., 11,169. In the female group, the trend between 2010 and 2019 was stable (*p* = 0.7321). The difference between the number of deaths recorded in 2020 and the average number of deaths recorded in the years 2017–2019 was calculated. It corresponded to a 6.3 % higher value (Table 2).

Between 2010 and 2015, death rates due to traffic accidents demonstrated a decreasing trend in both gender groups (Figure 1). This decline slowed down in 2015 and since that year trends were stable (*p* = 0.6438) (Table 2). Therefore, the arithmetic mean of the number of deaths which occurred in the years 2017–2019 was used to assess changes in the number of deaths in 2020, instead of the expected value calculated on the basis of the regression equation. The number of deaths observed in 2020 was lower than the average number by only four deaths. In the male group, the number of deaths due to accidents was 2.4 % higher; in the female group, the value was 8.4 % lower than the average, observed in the years 2017–2019.

Suicide-related mortality decreased between 2010 and 2019. CDRs decreased from 16.5 in 2010 to 11.9 in 2019 (APC = −4.7%, *p* = 0.0001). According to the regression model, the number of suicides in 2020 should have been 4,047. In fact, 506 more deaths were observed, i.e., 4,553 (12.5% increase). The differences were observed for both genders. In the male group, instead of the predicted 3,505 suicide deaths, 3,953 deaths were observed (12.8% increase). In the female group, 542 deaths were predicted, whereas 600 deaths occurred, which indicated that the number was 10.8% higher than expected.

A very rapid increase in mortality due to mental and behavioral disorders was observed between 2010 and 2019. The APC value due to these causes was 11.7% (*p* = 0.0015) (10.8%, *p* = 0.0029 in the male group and 15.7%, *p* = 0.0002 in the female group). The number of deaths expected in 2020 was 4,094 and the number of deaths which occurred was 4,387, which means it was 293 higher. The excess deaths due to this group of causes were observed in the male group (341 deaths, 10.8% increase). In the female group, mortality was lower than expected by 5.1%, i.e., 47 deaths.

## Discussion

The COVID-19 pandemic caused a significant increase in the mortality of the Polish population. In 2020, the recorded number of deaths was higher than the one expected on the basis of the trend determined for the years 2010–2019 by 15.5%, which corresponded to 64,037 deaths. In another study from Poland the excess deaths in 2020 were estimated compared to the average number of deaths in the 3 years preceding the COVID-19 pandemic. The excess mortality calculated with this method was 16.7% (8).

These excess deaths occurred mostly due to COVID-19 (62.5% of overall excess deaths). The remaining increase in mortality could have been related to: unreported COVID-19 deaths, deaths from inadequate access to healthcare services, and changes in disease patterns. A study conducted on 22 countries, excluding Poland, revealed that the number of deaths attributed to COVID-19 was underestimated by at least 35% (9).

The largest absolute increase of almost 10,000 deaths was reported for cardiovascular diseases. It was reported that since the year 1991, this group of diseases has been contributing less and less to mortality of the Polish population (10). It is supposed that the extended lifespan of the Polish population, i.e., by 51% in men and by 61% in women, observed after 1991, was directly associated with decreased mortality contributed by cardiovascular diseases (11). Due to progressive aging of the population, this decrease relates to standardized rather than crude death rates. However, our study showed that also crude death rates demonstrated a decreasing trend between the years 2010 and 2019, while the number of deaths increased by 5.9 % in 2020 compared to the expected value. Cardiovascular diseases are the main cause of death in the oldest age groups, and these people are at highest risk of rapid health deterioration due to limited or no access to medical care. A study conducted in England and Wales revealed an increase in the number of deaths in private homes, far above average values which were observed in previous years. It is estimated that the majority of these people may require care in late life. Emergency department attendance was 57% lower in April 2020 than in April 2019 (12). The United States also saw an increase in cardiovascular mortality in 2020, possibly partly related to misclassification of deaths due to COVID-19 and partly to delay in seeking by or receiving treatment in people with cardiovascular diseases (13, 14).

Mental and behavioral disorders also contributed to a significant increase in mortality in Poland in the year 2020. Mortality due to these causes increased between 2010 and 2019 at a rapid annual rate of 11.7%. In 2020, there was a further increase of 7.2% above the estimated number of deaths. However, it should be noted that this increase was due to a 10.8% higher male mortality rate, as the number of deaths in the female group was 5.1% lower than expected. As many as 95% of these deaths in the male group and 65% in the female group were due to alcohol use (according to the ICD-10, coded as F10). Between 2002 and 2017, annual alcohol consumption in Poland increased from 6.9 L to almost 10 L of pure alcohol per person. An increase in mortality, entirely attributable to alcohol consumption in Poland for both men and women and in all ages, was also observed during this period (15). The COVID-19 pandemic appears to have exacerbated the problem of excessive alcohol consumption that has existed for many years. It is also important to bear in mind a possible impact of limited access to therapy and treatment of people addicted to alcohol.

The COVID-19 pandemic had also a particular impact on people with Alzheimer's disease and other dementias. Studies have shown that even after taking into account old age and comorbidities, such as hypertension and diabetes, people with dementia are more susceptible to COVID-19 infection than those without dementia. Besides, older people with dementia, particularly those living in nursing homes, are at high risk of increased psychiatric symptoms and severe behavioral disorders being a result of social isolation during the pandemic (16–18). A study conducted in Poland during the second wave of the epidemic in November and December 2020 revealed that more than 20% of the respondents had symptoms of anxiety disorders and almost 19% had anxiety and depressive symptoms. Nearly 16% of respondents drank in a risky or harmful way, while more than 24% of respondents admitted to having experienced suicidal thoughts since the onset of the pandemic (19).

There has been a steady decline in suicide-related mortality in Poland since the year 2000 (20). Unfortunately, our own study showed that in the year 2020, an increase above the expected value of 12.5% (12.8% among men and 10.8% among women) was observed. The previously mentioned excessive alcohol consumption and psychological consequences of isolation are probably the reason for the increased number of suicidal deaths (21). In addition, a link between suicide and economic problems associated with the pandemic is also highlighted in literature (22).

Digestive diseases are also significantly caused by excessive alcohol consumption. Mortality caused by these diseases was increasing in the total population in Poland between 2010 and 2019. This was contributed by an increase in the number of male deaths (in the female group, changes in the trend were statistically insignificant). However, in 2020, the number of deaths was higher in comparison to expected deaths in both sex groups. In our study, we observed that alcoholic liver disease (K70) was responsible for 31% of deaths in this group of causes in 2020, while fibrosis and cirrhosis (K74), largely caused by alcohol consumption, accounted for a further 12% of deaths due to digestive diseases. These values were slightly higher than in previous years (23).

Mortality from respiratory diseases is rapidly increasing in both sex groups in Poland. However, in 2020, the male group demonstrated a 5.3% increase in deaths in comparison to the expected value, whereas in the female group, the number of deaths was 3.8% lower than expected. Chronic obstructive pulmonary disease (COPD) is responsible for ~20% of deaths due to respiratory diseases. A study carried out in Sweden showed that COPD patients hospitalized for acute COVID-19 disease were affected by significantly more comorbidities and were at high risk of severe outcome and death within 30 days (24).

What is undoubtedly noteworthy is a lower-than-expected number of deaths from cancer recorded in 2020, considering the fact that between 2010 and 2019, mortality from these causes was increasing in both gender groups. This may be caused by mistakenly classifying cancer deaths accompanied by a concurrent history of COVID-19 to this disease. Such coding is in line with WHO recommendations prioritizing code U07.1 in the sequence of events leading to death (25). Similar patterns were noted in studies conducted in Italy (26, 27). Determination of the actual number of cancer deaths in 2020 will in future require additional analyses with the use of the medical history of the deceased (28). In the future, it will also be possible to determine the scale of increased cancer mortality contributed by negligence in conducting preventive examinations and setting late diagnoses.

A very small difference between the number of deaths due to traffic accidents observed in 2020 in comparison to the average number of deaths for the years 2017–2019, when only 4 deaths were observed, seems slightly surprising. It would seem that due to periodic lockdowns, the number of deaths due to traffic accident should be lower than in previous years. However, this hypothesis proved true only in the female group (8.4% decrease). In the male group, the number of deaths increased by 2.4% in 2020. Results of a literature review on traffic accidents worldwide during the COVID-19 pandemic indicated that in 2020, 33 out of 42 countries experienced a decrease in traffic fatalities in comparison to the year 2019, while nine countries experienced increased mortality (29). The increase in the number of accident deaths in some countries is explained by a significant reduction of traffic and empty roads, which encourages exceeding speed limits (30–33).

A comparative analysis of excess mortality between the male and female groups shows that, in each of the analyzed cause-of-death groups, a higher increase (or, for malignant neoplasms, a smaller decrease) was observed in men. The difference between male and female life expectancy in Poland was 7.68 years in 2019 (81.75 for women and 74.07 for men) and 8.10 in 2020 (80.71 for women and 72.61 for men) (34). Studies analyzing mortality in populations exposed to extreme conditions such as famine, epidemics or slavery have shown huge potential of female survival even if mortality is extraordinarily high (35). Poland's experience in the 1st year of the COVID-19 pandemic seems to confirm this thesis.

### Limitations

There are several limitations of this study. The quality of analyses based on death statistics depends on the completeness and reliability of information contained in death certificates. Death records in Poland are fully complete. However, the quality of coding of death causes is not satisfactory, but at this moment, there are no better source of the data in Poland.

During the COVID-19 pandemic, proper coding of deaths became even more difficult as physicians confirming death did not always follow the WHO recommendations to prioritize U07.1 in the sequence of events leading to death. There might be plenty of reasons for such situation like physician mistake or omission (due to overwork), inability to test every patient for COVID-19, and finally, time lag between infection, recovery, and cause of death (8).

As a result, it is very difficult to determine what percentage of excess deaths were actually caused by COVID-19 and what percentage was caused by other reasons, such as resignation from medical care due to lockdown or fear of infection, or difficulties in accessing specialists or family doctors. Regardless, most excess deaths would not have occurred without the COVID-19 pandemic, so most of these deaths are a direct or indirect consequence of the pandemic.

## Conclusions

The difference between the expected and observed number of deaths of the Polish population in 2020 indicates a need for further analyses of causes of excess mortality.

It is necessary to monitor the phenomenon in subsequent years, aiming to identify permanent changes in the mortality pattern of the Polish population caused by the COVID-19 pandemic. However, a full understanding of the impact of the COVID-19 pandemic on human life will require additional analyses using medical records and medical histories of the deceased.

Identification of conditions and risk factors being causes of excess mortality will enable, in the future, in the event of further epidemics, to better focus on people at risk, which may result in better management of existing health care resources.

Comparing excess mortality in the years of the pandemic in Poland and other countries will allow for the assessment of strategies for limiting and mitigating the effects of the pandemic, also in terms of the ability and readiness of health care systems to treat COVID-19 patients and adapt to the challenges of the pandemic.

## Data availability statement

The raw data supporting the conclusions of this article will be made available by the authors, without undue reservation.

## Ethics statement

The studies involving human participants were reviewed and approved by the guidelines of the Declaration of Helsinki, and approved by the Bioethics Committee of the Medical University of Lodz on 22 May 2012 No. RNN/422/12/KB. Written informed consent for participation was not required for this study in accordance with the national legislation and the institutional requirements.

## Author contributions

MP contributed to study design, conducted the statistical analysis, interpreted the data, and writing the article. MK conducted the statistical analysis. MB contributed to study design. All authors participated in the critical revision of the article and approved the final article.

## Funding

This research was funded by Medical University of Lodz, Department of Epidemiology and Biostatistics (project no 503/6-029-07/503-61-001-19-00).

## Conflict of interest

The authors declare that the research was conducted in the absence of any commercial or financial relationships that could be construed as a potential conflict of interest.

## Publisher's note

All claims expressed in this article are solely those of the authors and do not necessarily represent those of their affiliated organizations, or those of the publisher, the editors and the reviewers. Any product that may be evaluated in this article, or claim that may be made by its manufacturer, is not guaranteed or endorsed by the publisher.
